# Peroxisomes Are Required for Lipid Metabolism and Muscle Function in *Drosophila melanogaster*

**DOI:** 10.1371/journal.pone.0100213

**Published:** 2014-06-19

**Authors:** Joseph E. Faust, Arvind Manisundaram, Pavlina T. Ivanova, Stephen B. Milne, James B. Summerville, H. Alex Brown, Michael Wangler, Michael Stern, James A. McNew

**Affiliations:** 1 Department of Biochemistry and Cell Biology, Rice University, Houston, Texas, United States of America; 2 Department of Pharmacology and the Vanderbilt Institute of Chemical Biology, Vanderbilt University School of Medicine, Nashville, Tennessee, United States of America; 3 Department of Molecular and Human Genetics, Baylor College of Medicine, Houston, Texas, United States of America; University of Houston, United States of America

## Abstract

Peroxisomes are ubiquitous organelles that perform lipid and reactive oxygen species metabolism. Defects in peroxisome biogenesis cause peroxisome biogenesis disorders (PBDs). The most severe PBD, Zellweger syndrome, is characterized in part by neuronal dysfunction, craniofacial malformations, and low muscle tone (hypotonia). These devastating diseases lack effective therapies and the development of animal models may reveal new drug targets. We have generated Drosophila mutants with impaired peroxisome biogenesis by disrupting the early peroxin gene *pex3*, which participates in budding of pre-peroxisomes from the ER and peroxisomal membrane protein localization. *pex3* deletion mutants lack detectible peroxisomes and die before or during pupariation. At earlier stages of development, larvae lacking Pex3 display reduced size and impaired lipid metabolism. Selective loss of peroxisomes in muscles impairs muscle function and results in flightless animals. Although, hypotonia in PBD patients is thought to be a secondary effect of neuronal dysfunction, our results suggest that peroxisome loss directly affects muscle physiology, possibly by disrupting energy metabolism. Understanding the role of peroxisomes in Drosophila physiology, specifically in muscle cells may reveal novel aspects of PBD etiology.

## Introduction

Peroxisomes are small, spherical, single-membrane bound organelles found in almost all eukaryotic cells. Most biochemical pathways in peroxisomes involve lipid metabolism, including β-oxidation of fatty acids (FAs), α-oxidation of branched chain FAs, and ether lipid biosynthesis [1]. Peroxisomes also participate in reactive oxygen species (ROS) metabolism. Sources of ROS, such as oxidases that produce H_2_O_2_, and antioxidant enzymes, such as catalase, are present in peroxisomes [2].

### Peroxisome Membrane Biogenesis

The formation of a mature, metabolically active peroxisome depends on the activity of Peroxin (Pex) proteins [3]. Peroxisomes can form *de novo* from the endoplasmic reticulum (ER) or divide autonomously from pre-existing organelles. The membrane for the organelle must be defined and proteins must be inserted into the membrane and the matrix. Pex3 is a peroxisomal membrane protein (PMP) that is required for *de novo* production from the ER as well as for ongoing membrane protein import from the cytosol [4]–[8]. During *de novo* organelle biogenesis, newly synthesized Pex3 localizes to the ER, concentrates into foci, and is carried away from the ER in buds that mature into peroxisomes [4]. Yeast, flies, and human cells lacking Pex3 display a complete block in peroxisome biogenesis [9]–[11]. In addition to the *de novo* biogenesis pathway, existing peroxisomes can grow and divide [12].

### Membrane and Matrix Protein Import

The import of proteins into the membrane and matrix of the peroxisome is a highly orchestrated process involving the action of many Pex proteins [13]. Two models exist for the insertion of peroxisomal membrane proteins (PMPs). In the classical model, Pex19 chaperones newly synthesized, cytoplasmic PMPs to the peroxisome and facilitates their insertion into the peroxisomal membrane [14], [15]. An alternative model suggests that all PMPs first insert in the ER membrane and are carried in buds that mature into peroxisomes [16]. These two pathways are not mutually exclusive, and most likely, both are utilized. The majority of Pex proteins are dedicated to the import of proteins into the peroxisomal matrix via two defined pathways. Proteins containing a peroxisome targeting signal (PTS) type 1 (PTS1), consisting of the C-terminal tripeptide SKL or other selected variants [17], are bound by cytoplasmic Pex5, shuttled to the peroxisome, and transported across the membrane [18]. A few proteins contain an N-terminal PTS type 2 (PTS2) motif that is recognized by the Pex7 receptor, and transported into the peroxisome in a similar fashion to PTS1-containing proteins [19]. The PTS2 system is not conserved across species. *D. melanogaster* and *C. elegans* appear to have lost the PTS2 system entirely and rely solely on the PTS1 import pathway [20], [21]. In addition to its roles in membrane biogenesis and PMP insertion, Pex3 has been implicated in pexophagy and peroxisome inheritance during cytokinesis [22], [23].

### Peroxisomes and Human Health

Impaired peroxisomal membrane biogenesis or protein import, caused by mutations in various *pex* genes, lead to a wide spectrum of human diseases, known collectively as peroxisome biogenesis disorders (PBDs) [24]. Peroxisome dysfunction disrupts many biochemical pathways and affects almost every organ system. In Zellweger syndrome (ZS), patients display defects in neuronal development, craniofacial malformations, and low muscle tone (hypotonia). Neuronal dysfunction is clearly present in PBD patients and is thought to be responsible for the observed hypotonia. However, myopathy has also been observed in PBD patients [25], [26], suggesting that muscles may be sensitive to peroxisome loss independent of neuronal involvement. There is no cure or effective therapy for ZS and afflicted patients usually die in infancy. The etiology of ZS is still unclear, but accumulation of very long chain FAs (VLCFAs) and low levels of ether lipids have been suggested to play causative roles [24].

### Animal Model Systems

Mice with impaired peroxisome biogenesis have been generated and serve as useful animal models for PBDs [27]. These animals show many phenotypes similar to PBD patients, including early death, VLCFA accumulation, neuronal migration defects, and locomotor problems. Drosophila have recently become an attractive system to study peroxisome biology and potentially model PBDs. The majority of human peroxisomal metabolic enzymes have homologs in *D. melanogaster* [20]. Three recent studies have shown that mutations in multiple Drosophila *pex* genes produce some phenotypes shared with mouse models and PBD patients [10], [28], [29].

In order to design effective therapeutics for PBDs, more mechanistic details are needed regarding the effects of peroxisome disruption on whole animal physiology. To this end, we have begun to characterize the effects of disrupting early peroxisome biogenesis on Drosophila physiology. Peroxisome biogenesis can be inhibited by a chromosomal mutation in *pex3* or by RNAi-mediated reduction in *pex3* mRNA. In the absence of Pex3, flies exhibit no detectable peroxisomes and show impaired lipid metabolism. Longer acyl chain triacylglycerols (TAGs) are elevated and the level of ceramide-phosphoethanolamine (CerPE), the fly analog of the essential lipid sphingomyelin, is reduced in *pex3* mutants. Flies with impaired peroxisome biogenesis specifically in muscle tissues show impaired muscle function, possibly due to altered energy metabolism. Human *PEX3* expression fully rescues peroxisome loss in the *pex3* deletion mutant. These flies containing “humanized” peroxisomes will be a useful model for examining pathological mutations in human PEX3.

## Results

### Peroxisome Distribution in Drosophila

Drosophila peroxisomes have been visualized previously in various tissues [10], [28]–[33]. We expanded the tissues examined, focusing primarily on larval tissues involved in energy metabolism: the gut, fat body, oenocytes, body wall muscles, and the epidermis. Nutrients are absorbed from the diet as they travels through the gut. Excess energy is stored as triacylglycerol within lipid droplets in the fat body, similar to adipose tissue in mammals. Depending on metabolic demands, lipids are mobilized from the fat body and are likely broken down in oenocytes, hepatocyte-like cells [34]. Larval body wall muscles have intensive energy demands, provided partially through lipid metabolism. Epidermal cells line the larval body wall between the cuticle and muscles.

**Figure 1:**
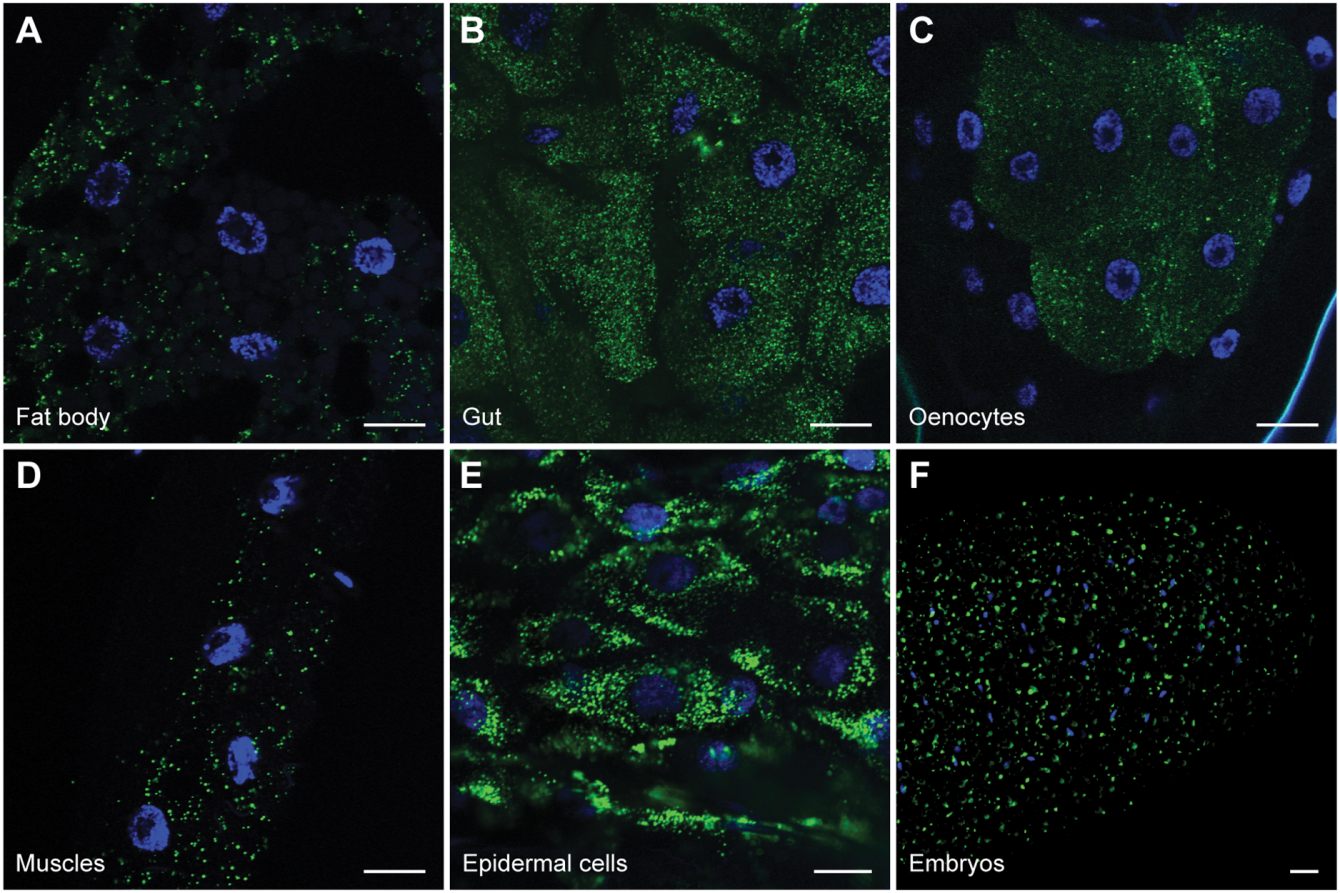
Peroxisome distribution in *D. melanogaster*. UAS-eYFP-PTS1 was expressed in various tissues and imaged in fixed samples by confocal microscopy. Peroxisomes are present in all tissues examined including, (**A**) Fat body (*lsp*>*eYFP-PTS1*), (**B**) Gut (*act5c*>*eYFP-PTS1*), (**C**) Oenocytes (*BO*>*eYFP-PTS1*), (**D**) Muscles (*24B*>*eYFP-PTS1*), and (**E**) Epidermal cells (*act5c*>*eYFP-PTS1*). (**F**) Embryos expressing PMP34-Cerulean were imaged by epifluorescence microscopy. Scale bar = 20 µm.

A peroxisomal matrix marker was generated by appending the PTS1 targeting signal, –KNPPETKSKL, of Carnitine O-acetyltransferase (CRAT; CG1041) on enhanced Yellow Fluorescent Protein (eYFP-PTS1) and tissue specific expression was achieved by means of the GAL/UAS system [35]. eYFP-PTS1 localizes to the matrix of peroxisomes, which are visible as punctae in the cytoplasm. All tissues examined, including the gut (Fig. 1B), fat body (Fig. 1A), oenocytes (Fig. 1C), muscles (Fig. 1D), epidermal cells (Fig. 1E), and early embryos (Fig. 1F), contain abundant peroxisomes. Some cell types, such as oenocytes and gut, contain more peroxisomes than other tissues.

### Characterization of *pex3* Mutants

**Figure 2:**
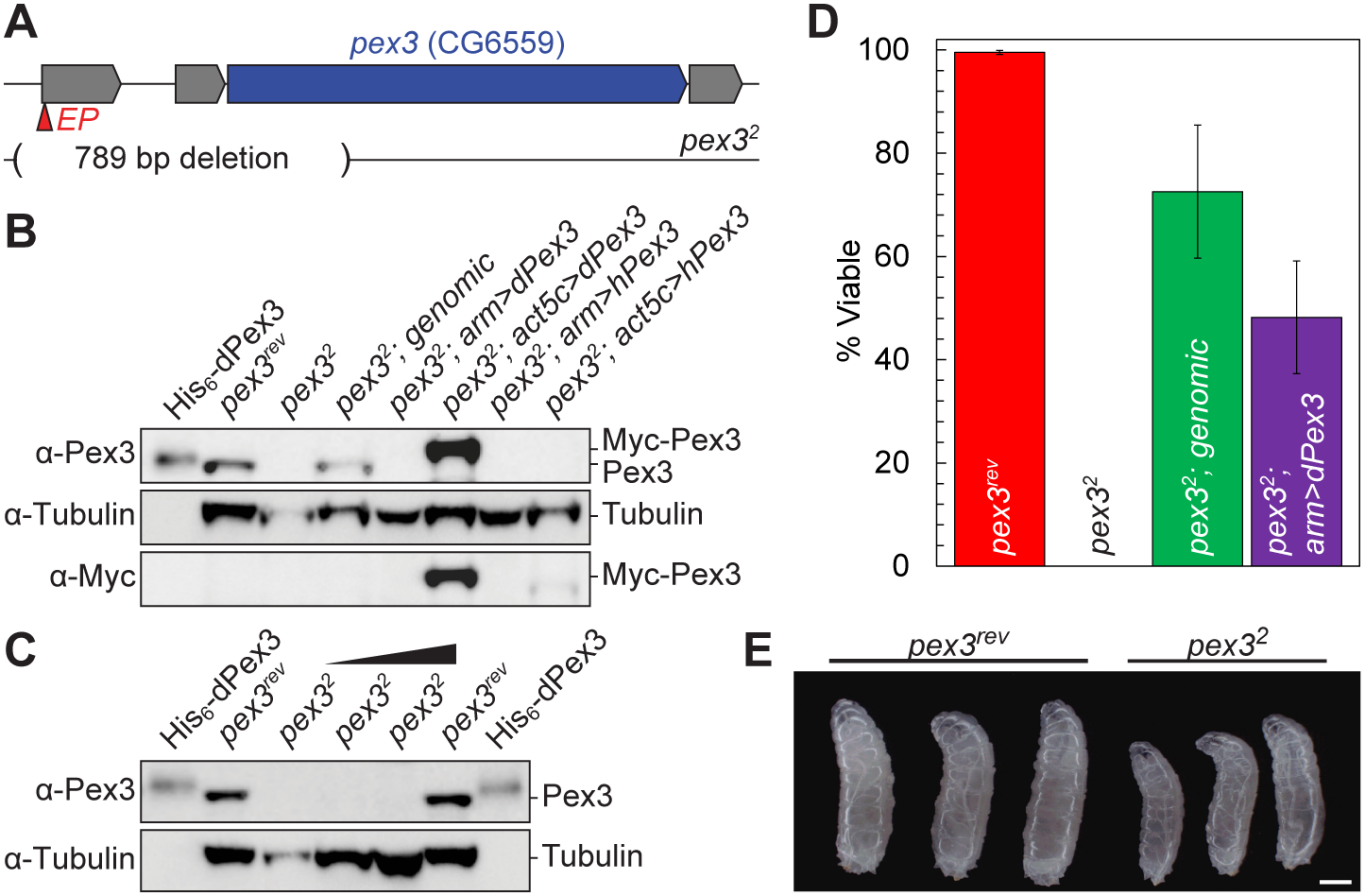
*pex3^2^* is a null mutant. (**A**) A schematic of the pex3 genomic region showing the EP element insertion site and the deleted region in *pex3^2^*. (**B**) Immunoblot of total protein extracts from wandering third instar larvae show that Pex3 protein is undetectable in the *pex3^2^* mutants, but can be detected by the addition of a genomic fragment containing *pex3* or expression of a fly or human cDNA transgene. The Myc-tagged transgenic protein is a higher molecular weight than endogenous Pex3. 37.5 µg of total protein was loaded in lanes 2–8. (**C**) Immunoblot with increasing amounts of *pex3^2^* protein shows that Pex3 protein is undetectable. Total protein amounts loaded in lanes 2–6, were 37.5 µg, 37.5 µg, 75 µg, 112.5 µg, and 37.5 µg. The intensities of the Tubulin bands in lanes 3–6, relative to lane 2, are 30%, 94%, 124%, and 108%.(**D**) Adult fly viability measurements show that the loss of pex3 (*pex3^2^*) results in nonviable Drosophila 0±0% (n = 381) compared to controls (*pex3^rev^*; 99.5±0.4%; n = 472), genomic rescue (72±12.9%; n = 185), and fly cDNA rescue (48.2±10.9%, n = 46). (**E**) Imaging of wandering third instar larvae shows that *pex3^2^* larvae are smaller than *pex3^rev^* control larvae.

In order to inhibit peroxisome biogenesis, we chose *pex3* as our target for mutagenesis. Pex3 is involved in the early steps of *de novo* biogenesis of peroxisomes from the ER and yeast, flies and human cells lacking *pex3* have no detectable peroxisomes [9]–[11]. We generated a pex3 deletion mutant by mobilizing a P-element transposon (P{EPgy2}Pex3^EY19206^) in the 5′ untranslated region of the *pex3* gene and screening for imprecise excision events. One imprecise excision allele, *pex3^2^*, carries a 789 bp deletion containing the first non-coding exon, intron one, and part of exon two including the translational start site and 285 bp of the coding sequence (Fig. 2A). We used a precise excision of the P-element, *pex3^rev^*, as well as genomic and cDNA rescue transgenes as controls for phenotypic analysis. *pex3^2^* mutants reared on standard cornmeal/agar died before the wandering third instar larval stage (Fig. 2D). The larval lethality failed to complement a chromosomal deficiency Df(3L)BSC837, which uncovers the *pex3* locus [36]. The *pex3^2^* lethality also failed to complement the previously reported *pex3^1^* deletion allele [10]. Genomic and cDNA rescue significantly improved viability of the *pex3^2^* mutant (Fig. 2D), but did not fully rescue indicating that expression levels or tissue specific expression may not have been fully recapitulated. Additionally, *pex3^2^* mutant larvae are smaller than age-matched *pex3^rev^* control larvae (Fig. 2E). When grown on grape juice plates, most *pex3^2^* flies survived to the pupal stage and some escaper adults were observed. These few adult escapers were severely disabled, could not fly, and died within several days.

To determine if *pex3^2^* makes a protein product, we generated anti-Pex3 antibodies in rabbits using recombinant Pex3- His_6_ protein. Anti-Pex3 serum recognizes recombinant Pex3-His_6_ and endogenous Pex3 protein in total protein extracts from *pex3^rev^* larvae (Fig. 2B), yet no Pex3 band is visible in extracts from *pex3^2^* larvae (Fig. 2B and 2C). The genomic *pex3* rescue fragment was sufficient to restore Pex3 protein to detectable levels, similar to wildtype, in *pex3^2^* (Fig. 2B). Pex3 protein is not detectable by immunoblotting when a *UAS-dPex3-Myc* transgene was driven by the weak global driver, *arm-GAL4* (Fig. 2B). However, the same transgene driven by the strong, global driver, *act5c-GAL4*, produces levels of Pex3 protein higher than endogenous levels (Fig. 2B). Similarly, *act5c-GAL4* driving human *pex3* transgene (*UAS-hPex3-Myc*) also produced Pex3 protein, detectable by anti-Myc antibodies, but at levels lower than Drosophila *pex3* (Fig. 2B).

### *pex3^2^* Mutants Lack Peroxisomes

**Figure 3:**
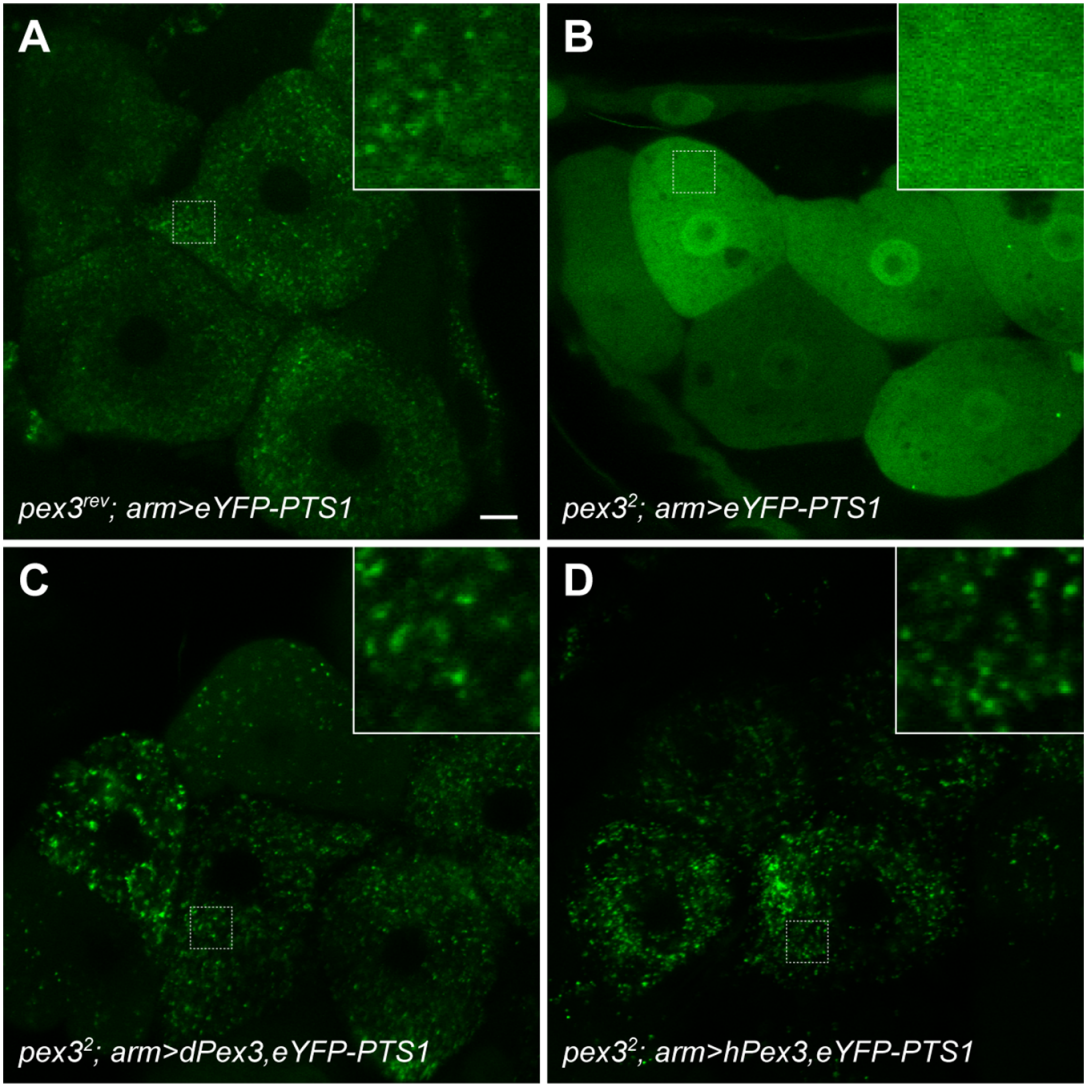
Peroxisome biogenesis is impaired in *pex3^2^* mutants. Peroxisomes were visualized by confocal microscopy in hepatocyte-like, oenocyte cells of third instar larvae. *UAS-eYFP-PTS1* was driven by the *arm* GAL4 driver in various backgrounds. (**A**) Peroxisomes are abundant in oenocytes of *pex3^rev^* control larvae. (**B**) eYFP-PTS1 is diffuse in the cytoplasm of *pex3^2^* larvae indicating that peroxisomes are absent. Some cells contain bright spots, but it is unclear if these are peroxisomes. Peroxisomes are restored when fly (**C**) or human (**D**) *pex3* cDNA are expressed in the *pex3^2^* background. Scale bar = 10 µm.

We next examined the effect of Pex3 loss on peroxisome biogenesis. The peroxisomal matrix marker, *UAS*-*eYFP-PTS1*, was driven by the weak global *arm*-GAL4 driver and the hepatocyte-like oenocytes cells, were examined in wandering 3^rd^ instar larvae. Many peroxisomes were visible as punctae in the cytoplasm of oenocytes in *pex3^rev^* wandering 3^rd^ instar larvae (Fig. 3A), but no punctae are present and eYFP-PTS1 remains diffuse in the cytoplasm in the *pex3^2^* mutant (Fig. 3B). This result indicates that *pex3^2^* mutants lack peroxisomes that can import PTS1 cargo. Larvae expressing Drosophila (Fig. 3C) or Human (Fig. 3D) *pex3* cDNA in the *pex3^2^* genetic background recover cytoplasmic punctae, indicating the presence of peroxisomes. These results demonstrate that the impaired biogenesis in *pex3^2^* is due to the loss of *pex3* and that Human Pex3 can replace the function of Drosophila Pex3 and restore peroxisome biogenesis.

### *pex3^2^* Mutants have Altered Lipid Metabolism

**Figure 4:**
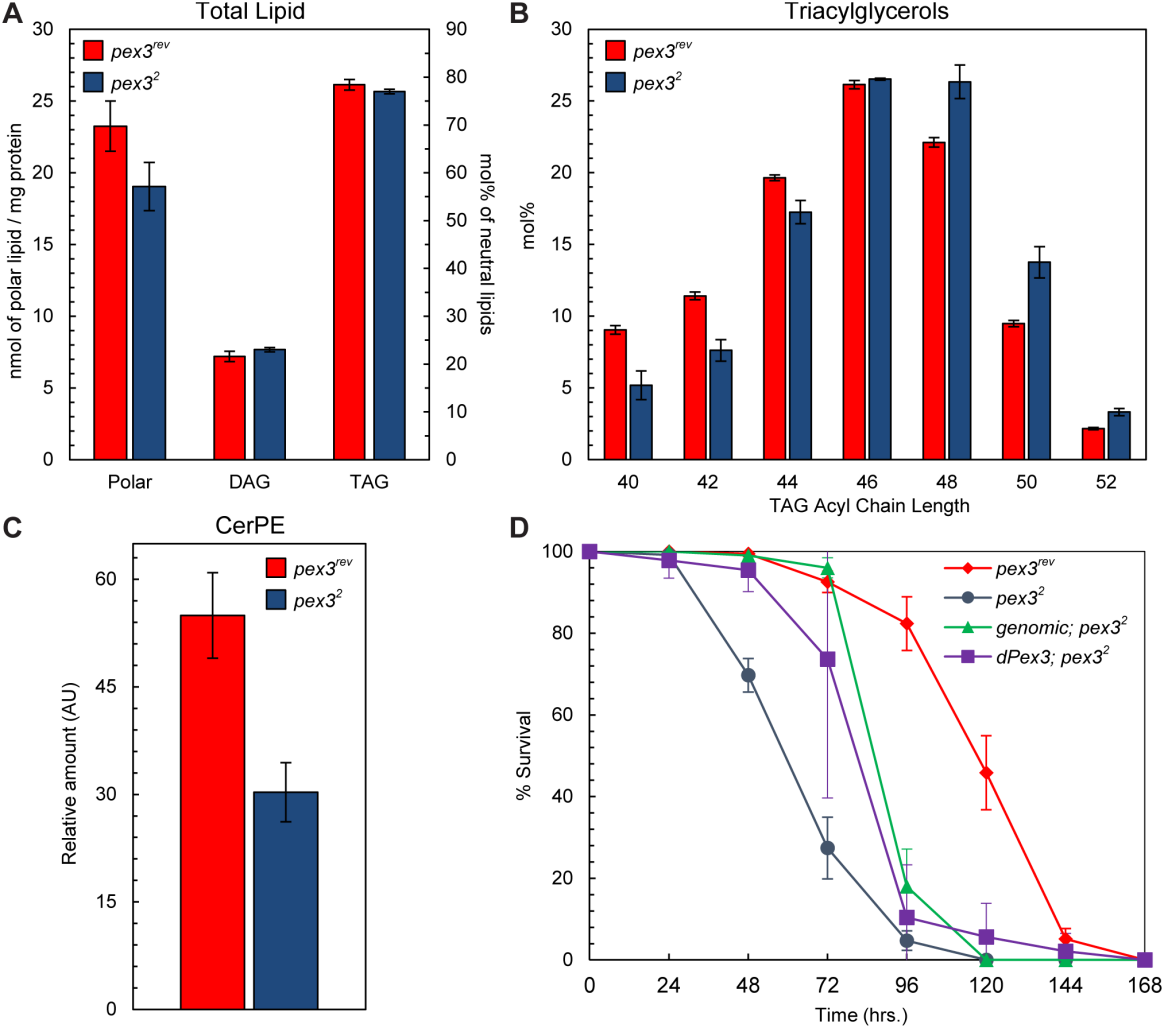
Peroxisome loss causes aberrant lipid metabolism. (**A**) Mass spectrometry (MS) analysis of larval lipids shows that the levels of the major lipid classes, Polar lipids, DAG, and TAG are unchanged in *pex3^2^* mutants. (**B**) However, *pex3^2^* mutants have elevated longer acyl chain length TAG species. (**C**) CerPE levels are decreased in *pex3^2^*. (**D**) Survival analysis of larvae under starvation conditions reveals that larvae lacking Pex3 (*pex3^2^*) are hypersensitive to starvation. Starvation sensitivity can be partially rescued by expression of genomic *pex3* or *pex3* cDNA.

**Figure 5:**
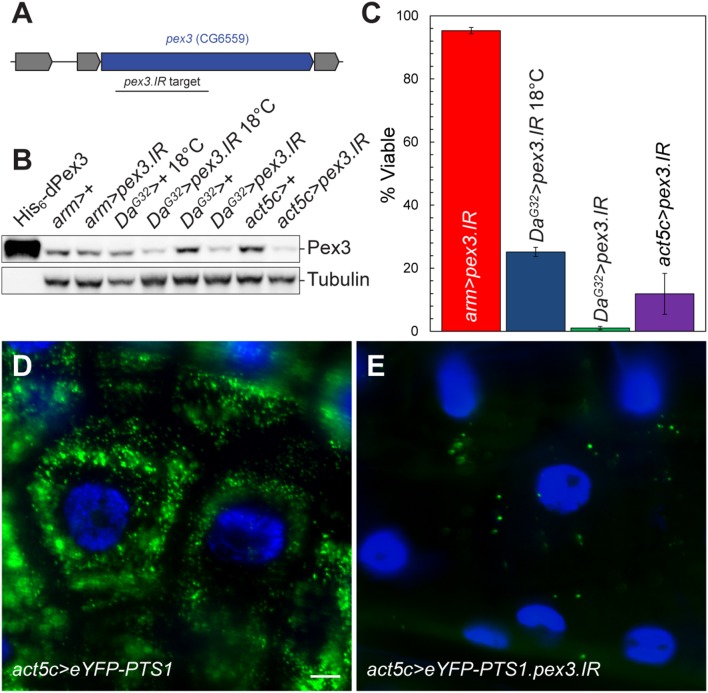
RNAi knockdown of pex3 reduces peroxisome numbers and impairs viability. (**A**) A schematic of the pex3 genomic region shows the area targeted by the inverted repeat. (**B**) Immunoblot of total protein extracts from wandering third instar larvae shows that Pex3 protein levels are reduced when *UAS-pex3.IR* is driven by global GAL4 drivers. The weak, global driver armadillo (*arm*) driving *UAS-pex3.IR* does not detectably reduce Pex3 levels. The medium strength global driver, daughterless (*Da^G32^*), driving *UAS-pex3.IR* reduces Pex3 levels at 18°C and room temperature. The strong global driver, actin 5c (*act5c*), driving *UAS-pex3.IR* reduces the level of Pex3 even further. (**C**) Adult viability of flies expressing pex3.IR driven by global GAL4 drivers was measured. Weak knockdown (*arm*>*pex3.IR*) did not appreciably decrease viability (95.3±1.0%; n = 190). At 18°C, viability of *Da^G32^*>*pex3.IR* flies was reduced to 25.2±1.5% (n = 330). At room temperature, viability of *Da^G32^*>*pex3.IR* flies fell to 1±0.5% (n = 444). At both temperatures, some flies died as pupae or pharate adults and remained trapped in the pupal case. Viability of act5c>*pex3.IR* flies was also reduced (11.9±6.5%; n = 106). (**D**) Microscopy of wandering third instar larval epidermal cells shows that peroxisome numbers are severely reduced when UAS-pex3.IR is driven by the act5c GAL4 driver compared to controls lacking the RNAi transgene (**E**).

The major biochemical pathways in peroxisomes involve lipid metabolism, such as β-oxidation of FAs, ether lipid synthesis, etc. We predicted that lipid metabolism would be altered if peroxisome biogenesis was blocked and tested this prediction by performing lipidomic analysis on *pex3^2^* mutants using liquid chromatography-mass spectrometry (LC-MS). The abundance of major lipid classes, polar lipids, diacylglycerols (DAG), and TAG, were not significantly altered in the *pex3^2^* mutant compared to the *pex3^rev^* control (Fig. 4A). However, the composition of FAs in storage lipids (TAGs) were changed. Longer acyl chain length TAGs are elevated and shorter acyl chain length TAGs are reduced in *pex3^2^* (Fig. 4B). Similar changes in TAG chain length were observed in larvae with reduced *pex3* (Fig. S1). A UAS-inducible transgene (*UAS-pex3.IR*) encoding a hairpin RNA that targets *pex3* for degradation by RNAi (Fig. 5A) was used to reduce the level of Pex3. When the *UAS-pex3.IR* was driven by the global GAL4 driver, daughterless (*Da^G32^*), longer acyl chain length TAGs are elevated and shorter acyl chain TAGs are reduced (Fig. S1). In addition to these changes in TAG acyl chain length, we found that the insect specific lipid CerPE is significantly reduced in *pex3^2^* (Fig. 4C). Insects use CerPE in place of sphingomyelin, a sphingolipid important in neuronal membranes [37]. This result suggests a previously unknown contribution of peroxisomes to CerPE biosynthesis.

Altered TAG chain length raised the possibility that lipid metabolism might be defective in *pex3^2^*. This prompted us to examine the ability of the *pex3^2^* larvae to survive under starvation conditions, where lipid catabolism is required for survival. We found that *pex3^2^* mutants are hypersensitive to starvation (Fig. 4D) suggesting that peroxisomes, likely through β-oxidation of FAs, contributes to starvation-induced TAG breakdown and overall energy metabolism in the animal.

### Global Loss of *pex3*

Many tissues, including the nervous system, muscles, and liver are affected in PBD patients; however, tissue-specific peroxisomal function has not been significantly examined. We selectively inhibited peroxisome biogenesis in specific tissues by RNA interference to uncover tissue specific roles. Peroxisomal loss, accomplished by *pex3* knockdown, was first examined in all tissues for comparison with the *pex3^2^* mutant. Global expression of *UAS-pex3.IR* decreased Pex3 levels dependent on the relative strength of the global GAL4 drivers (Fig. 5B). With the weak *arm-GAL4* driver, Pex3 levels are slightly reduced from the control (76% of *arm>+*). The intermediate strength *Da^G32^-GAL4* driver reduces Pex3 levels to 24% of wildtype (at both 18°C and 23°C). The strong *act5c-GAL4* driver further reduces Pex3 levels to 14% of wildtype. Peroxisome abundance, revealed by the peroxisomal matrix marker eYFP-PTS1, is also reduced when *UAS-pex3.IR* is driven by *act5c-GAL4* (Fig. 5E) compared to controls lacking *UAS-pex3.IR* (Fig. 5D).

Viability of *pex3* knockdown larvae was positively correlated with Pex3 levels. *arm>pex3.IR* flies have 95.3% ±1.0% viability while *act5c>pex3.IR* flies have 11.9% ±6.5% viability. *Da^G32^>pex3.IR* viability is very low, 1.0% ±0.5% at 23°C, but increased to 25.2% ±1.5% at 18°C (Fig. 5C), presumably a consequence of the known temperature dependence of GAL4. However, the Pex3 levels in *Da^G32^>pex3.IR* flies at 23°C and 18°C and indistinguishable by immunoblotting (Fig. 5B) suggesting that undetectably small differences in Pex3 protein levels can have substantial effects on viability.

### Tissue-specific Loss of *pex3*

**Figure 6:**
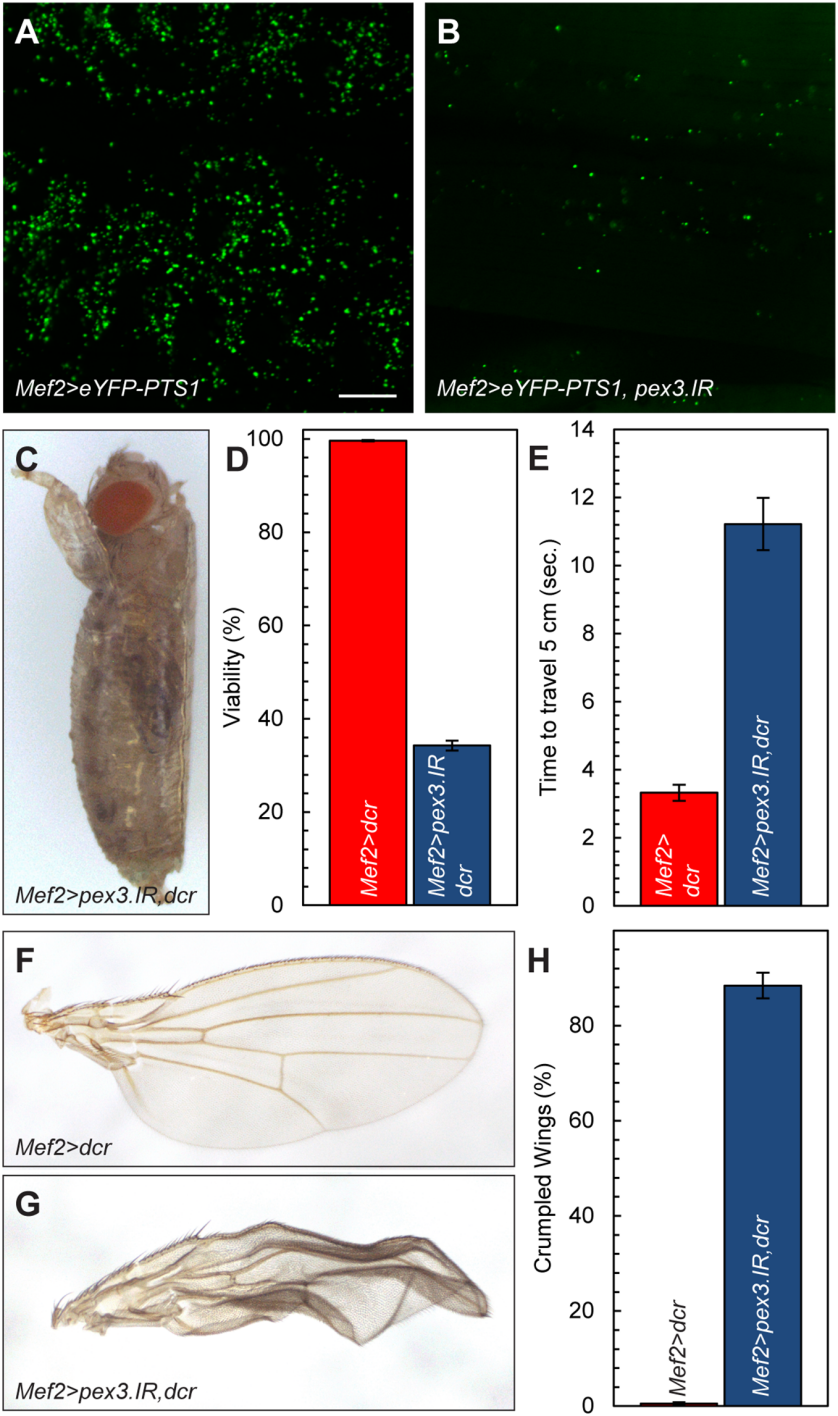
Peroxisome loss causes muscle defects. *pex3* knockdown in wandering third instar larval body wall muscles (**B**, *Mef2*>*eYFP-PTS1*, *pex3.IR*) leads to a reduction in muscle peroxisome numbers compared to controls (**A**, *Mef2*>*eYFP-PTS1*). (**C**) *pex3* knockdown in muscles (*Mef2*>*pex3.IR*,*dcr*) causes some flies to die as fully formed, pharate adults trapped in the pupal case. (**D**) Viability of *Mef2*>*pex3.IR*,*dcr* flies is reduced to 34.1±1%. (**E**) Climb tests of adult flies with reduced pex3 in muscles shows that *Mef2*>*pex3.IR*,*dcr* flies display impaired locomotor ability, as measured by the time required to climb 5 cm (11.2±0.8 sec) compared to controls (3.3±0.2 sec). Some *Mef2*>*pex3.IR*,*dcr* flies (**G**) have “crumpled” wings compared to *Mef2*>*dcr* control flies (**F**), possibly due to impaired muscle function required for wing expansion. (**H**) Of the flies that successfully eclose, 88.4±2.7% have crumpled wings. Scale bar = 20 µm.

We next expressed the *pex3* RNAi transgene in a variety of tissues and found that muscles are sensitive to the loss of peroxisomes when screened for viability (Table 1). Peroxisome numbers are severely reduced in larval body wall muscles (Fig. 6B) when *UAS-pex3.IR* is driven by the *Mef2-GAL4* muscle driver compared to controls lacking *UAS-pex3.IR* (Fig. 6A). Stronger *pex3* knockdown in muscles, achieved by co-expression of the RNAi component Dicer (Dcr), resulted in lethality at the pharate adult stage (Fig. 6C). Most of these pharate adults had begun eclosing as indicated by the open operculum, the opening in the pupal case through which the adult emerges. Viability of *Mef2>pex3.IR,dcr* is thus reduced to 34% ±1% compared to 99.6% ±0.2% for *Mef2>dcr* controls (Fig. 6D). Wing expansion is also significantly impaired in *Mef2>pex3.IR,dcr* flies (Fig. 6G) compared to *Mef2>dcr* controls (Fig. 6F). Of the *Mef2>pex3.IR,dcr* flies that successfully eclose, 88% ±3% have crumpled wings compared to 0.5% ±0.3% for *Mef2>dcr* controls (Fig. 6H). Finally, *Mef2>pex3.IR*,dcr decreases locomotion, as determined by a climb test, in which the time required to crawl 5 cm was measured. *Mef2>pex3.IR,dcr* animals took 11.2±0.8 seconds to crawl 5 cm while the *Mef2>dcr* controls needed only 3.3±0.2 seconds to travel the same distance (Fig. 6E and Video S1). Knockdown of *pex3* with another muscle driver, *24B-GAL4*, also results in trapped eclosion, crumpled wings, and decreased locomotion (Table 1 and data not shown). This suggests that the loss of peroxisomes specifically in muscles impairs locomotion and two processes that require muscle function, eclosion from the pupal case and wing expansion. The impaired locomotion observed in flies with reduced *pex3* is unlikely to be consequence of inappropriate innervation since evoked excitatory junctional potentials (EJPs) were normal in *pex3^2^* mutant larvae (Fig. S3). Additionally larval body wall muscle structure and synaptic bouton morphology at neuromuscular junctions appear normal in *pex3^2^* mutants (Fig. S2).

**Table 1:** Peroxisome loss causes pupal lethality and eclosion defects.

Driver	Tissue(s) affected	Viable larvae	Viable adults
*tub*	All, strong	+	−
*act5c*	All, strong	+	−
*Da^G32^*	All, strong	+	semi-lethal
*arm*	All, weak	+	+
*r^4^*	Fat body	+	+
*Lsp2*	Fat body	+	+
*BO*	Oenocytes	+	+
*elav*	Neurons	+	+
*D42*	Motorneurons	+	+
*OK6*	Motorneurons	+	+
*Ddc*	Dopaminergic and Serotinergic Neurons	+	+
*Gli*	Glia	+	+
*repo*	Glia	+	+
*amn^C651^*	Prothoracic Gland	+	+
*24B*	Muscles	+	semi-pharate lethal
*Mef2*	Muscles	+	semi-pharate lethal

## Discussion

Consistent with previous studies, we have found that peroxisomes are present in all tissues we examined in *Drosophila melanogaster* (Fig. 1), but the abundance of peroxisomes per cell is not uniform in all tissues [10], [28]–[33]. We have generated a *pex3* mutant that lacks detectable peroxisomes (Fig. 3B) and does not survive to adulthood (Fig. 2D), also consistent with a previously reported *pex3* allele [10]. Impaired peroxisome biogenesis specifically in muscles impairs muscle function (Fig. 6). Muscle dysfunction is also seen in PBD patients, but is thought to be a secondary consequence of neurological defects. We suggest that muscle function may require peroxisomal metabolism independent from any neurological effects.

### Lipid Metabolism

Alterations in lipid metabolism were also observed in *pex3^2^* mutants (Fig. 4). The primary biochemical pathway in peroxisomes is β-oxidation of FAs. In yeast and plants, all β-oxidation occurs in peroxisomes. In mammals, peroxisomes and mitochondria are metabolically linked and coordinate β-oxidation. FAs with longer acyl chain lengths are preferentially broken down in peroxisomes while medium and shorter chain FAs are preferentially broken down in mitochondria. There is likely a handoff of chain-shortened FAs from peroxisomes to mitochondria. The relative contribution of mitochondria and peroxisomes in Drosophila is unknown. It is likely that longer acyl chain FAs are broken down in peroxisomes since mutants defective in peroxisome biogenesis accumulate small amounts of VLCFAs [10], [28]. We also see elevated longer acyl chain TAGs and reduced shorter acyl chain length TAGs in *pex3^2^* mutants (Fig. 4B). VLCFAs are low abundance lipids and likely below the detection limit of the analysis described here. The lipids measured in our LC-MS analysis represent the major classes present in the larvae.

*pex3^2^* mutants are also hypersensitive to starvation (Fig. 4D). Excess nutrients are stored in the fat body primarily as TAG within storage organelles called lipid droplets. During starvation, lipids are mobilized from the fat body, circulate as DAGs in the hemolymph and are broken down by β-oxidation in target tissues [34]. Although mitochondrial β-oxidation genes are up-regulated during starvation [38], indicating an important role for mitochondrial β-oxidation in the starvation response, our data suggests that peroxisomes are also required for survival during starvation. Starved yeast display a similar requirement for peroxisomes; mutations in peroxisome biogenesis genes decrease yeast survival under starvation conditions [39]. This starvation sensitivity is associated with decreased lipid droplets as *pex3*Δ yeast have fewer, smaller lipid droplets [40]. The decrease in lipid droplets could reflect impaired lipid storage and could interfere with lipid mobilization. We did not observe a change in total TAG levels in *pex3^2^* mutants (Fig. 4A). However, whole larvae were used in our analysis and we cannot exclude the possibility that tissue-specific differences were present.

*pex3^2^* mutants die before the wandering 3^rd^ instar larval stage when reared on standard cornmeal/agar media. Altering diet, and likely nutrient availability, improves viability, but most flies still die as pupae and do not survive to adulthood. Additional studies using defined media will be required to identify the nutrient(s) responsible for this effect. Beginning with the wandering 3^rd^ instar stage, larvae are no longer consuming food and must rely on the metabolism of stored nutrients for metamorphosis. It is possible that peroxisomes are required for at least part of this metabolism. For example, there may be specific lipids that are poor substrates for mitochondrial β-oxidation and require peroxisomes for their degradation. In addition, peroxisomes might also be required for specific biosynthetic pathways required for larval and pupal development. For example, plants require peroxisomal β-oxidation to generate active forms of auxin and jasmonates [41], [42], raising the possibility that there may be specific *D. melanogaster* metabolites (e.g. hormones) that require peroxisomes for biosynthesis.

A surprising find in our lipidomics analysis was the reduction in CerPE levels in *pex3^2^* mutants (Fig, 4C). CerPE is used in insects as an analog of sphingomyelin (SM), which is important for neuronal membrane stability and lateral organization in mammals [37], [43]. The larval brain and wing imaginal disk have relatively high CerPE levels [44]. A recent study found that CerPE is critically important in glial cells, specifically in the wrapping of axon bundles [45]. Wrapping glia in Drosophila may play a similar role to myelination in mammalian, e.g. to insulate motor axons. The same group found that the knockdown of genes involved in peroxisome biogenesis (e.g., *pex10*), peroxisomal β-oxidation (e.g., bifunctional protein), and ether lipid synthesis (e.g., glyceronephosphate O-acyltransferase) in wrapping glia were among the 736 genes that caused locomotor defects similar to knockdown of CerPE biosynthetic genes [45]. The locomotor defects in these mutants may be directly caused by low CerPE levels. Neither CerPE nor SM is directly synthesized in peroxisomes [46]–[48] and the effect of peroxisome dysfunction on CerPE levels is likely indirect and remains to be discovered.

### Peroxisomes in Muscles

We have observed that knockdown of *pex3* specifically in muscles impairs multiple processes that require muscle function, eclosion (Fig. 6C), wing expansion (Fig. 6E), and climbing (Fig. 6H). These results were obtained using two muscle drivers, *Mef2* and *24B-GAL4*. *GAL4* expression is restricted to muscle cells in the *Mef2* driver line [49]–[51]. In the *24B* driver line, *GAL4* is expressed in somatic muscles and a subpopulation of neurons [35], [52]. The expression patterns of these drivers suggest that loss of *pex3* in somatic muscles is responsible for the observed phenotypes. However, we cannot rule out contributions from other tissues that express *GAL4* in these driver lines.

Our results are consistent with a previous study that showed *pex16* mutant adults have impaired locomotion and the degree of impairment worsens with age [10]. Impaired muscle function is seen in PBD patients and PBD animal models, but is thought to be a consequence of neurological defects [24], [25], [27], [53]–[55]. Our results suggest that peroxisomes are required for muscle function independent of neurological involvement.

In what ways might peroxisomes be required for muscle function? Peroxisomes may be directly involved in FA metabolism for energy production. If so, then longer acyl chain lipids may accumulate in muscle lacking peroxisome, which could lead to toxicity, specifically to mitochondria. Such FA toxicity could explain the defects in mitochondrial morphology and activity that have been observed in PBD patients and PBD mouse models [25]–[27], [53], [56]–[60]. Other peroxisomal substrates, such as bile acid intermediates and phytanic acid, can induce mitochondrial damage [61], [62]. Alternatively, the absence of peroxisomes may also increase ROS levels leading to mitochondrial damage [60]. Because peroxisomes and mitochondria are metabolically linked, breaking the connection between the organelles may have detrimental consequences for the cell.

### Conclusions

Through this study we have gained new insights into peroxisome biology in *D. melanogaster* and examined the effects of peroxisome loss on animal physiology. Peroxisomes are required for Drosophila development, possibly for their role in lipid breakdown during metamorphosis. Not surprisingly, we find alterations in lipid content when peroxisome biogenesis is impaired. Muscle function is also impaired when peroxisome numbers are reduced specifically in muscle cells. These results raise the possibility that the muscle defects seen in PBD patients may be due to problems within the muscle in addition to the known neurological defects. Exploring peroxisome function in Drosophila may have revealed a previously underappreciated role of muscle in the PBD disease state.

## Materials and Methods

### Drosophila Stocks

The following *GAL4* driver lines were obtained from the Bloomington Drosophila Stock Center at Indiana University (BDSC): *tub* (#5138), *act5c* (#3954), *arm* (#1560), *r4* (#33832), *Lsp2* (#6357), *elav* (#458), *repo* (#7415), *24B* (#1767), *Mef2* (#27390) and *Ddc* (#7009). The *Da^G32^* (#108252) *GAL4* driver line was provided by the Drosophila Genetic Resource Center. *BO GAL4* [34] was provided by Dr. Alex Gould. *OK6 GAL4* [63] was provided by Dr. Hermann Aberle. *D42 GAL4* [64] was provided by Dr. Thomas Schwarz. *Gli GAL4* [65] was provided by Dr. Vanessa Auld. *amn^C651^* [66] was provided by Dr. J. Douglas Armstrong. The BDSC also provided *UAS*-dcr (#24651), EP-Pex3 (#22152), and Dr1/TMS, P(Δ2–3)99B (#1610). The National Institute of Genetics (NIG) in Japan provided *UAS*-pex3.IR (#6859R-4).

All fly stocks were maintained on cornmeal/agar (6% dextrose, 6.8% cornmeal, 1.2% yeast, 0.72% agar, 2% nipagen) or cornmeal/molasses/agar (8% molasses, 6% cornmeal, 1.5% yeast, 0.6% agar, 1% nipagen, 0.75% propionic acid) Drosophila media at room temperature (23°C).

### Plasmid Construction

#### pJM573 (*UAS-eYFP-PTS1* in pUAST)

*eYFP* was PCR amplified using the oligos KpnI-eYFP (GCGGTACCATGGTGAGCAAGGGCGAG) and eYFP-PTS1-XbaI (GCTCTAGATTACAACTTCGACTTAGTCTCAGGCGGGTTCTTCTTGTACAGCTCGTCCATG). PCR product was digested with KpnI and XbaI and ligated into pUAST cut with the same enzymes.

#### pJM623 (Tub-dPMP34-cerulean in pCaSpeR 4-tubulin)

Cerulean was PCR amplified using the oligos NotI-GFP (GCGGCCGCAACCATGGTGAGCAAGG) and GFP-XhoI (CTCGAGTTACTTGTACAGCTCGTCC). Cerulean PCR product was cloned into pCR-Blunt II TOPO using a Zero Blunt PCR cloning kit (Invitrogen). Cerulean was cut from pCR-Blunt II TOPO using NotI and XhoI and ligated into pCaSpeR 4-Tubulin cut with the same enzymes. dPMP34 (CG32250) was PCR amplified from a Drosophila Genomics Resource Center (DGRC) cDNA clone (#RE36975) using the oligos KpnI-EcoRI-Dm_PMP34 (GGTACCGAATTCACAAAATGGTGGCCCCCTCG) and Dm_PMP34-NotI (GCGGCCGCGTTGCGCTTAAGCAGC). dPMP34 PCR product was cloned into pCR-Blunt II TOPO using a Zero Blunt PCR cloning kit (Invitrogen). dPMP34 was cut from pCR-Blunt II TOPO using KpnI and NotI and ligated into Cerulean in pCaSpeR 4-Tubulin cut with the same enzymes.

#### pJM875 (*UAS-dPex3-Myc* in pUAST attB)

5′ phosphorylated oligos, KpnI-NcoI-myc-XbaI-top (CCCATGGGAACAAAAACTTATTTCTGAAGAAGACTTGTAGT) and KpnI-NcoI-myc-XbaI bottom (CTAGACTACAAGTCTTCTTCAGAAATAAGTTTTTGTTCCCATGGGGTAC) were annealed and ligated into pUAST cut with KpnI and XbaI. dPex3 was PCR amplified from a cDNA clone (DGRC #LD41491) using the oligos BglII-pex3 2 (GCAGATCTATGCTGTCGCGCCTGC) and Dm_pex3-EagI (AACGGCCGAGCGGAGCTAAAGC). PCR product was cut with BglII and EagI and ligated into Myc in pUAST cut with the same enzymes.

#### pJM877 (*UAS-hPex3-Myc* in pUAST attB)

hPex3 was PCR amplified from a cDNA clone (ATCC #MGC-9125) using the oligos BglII-Hs_pex3 (GCAGATCTATGCTGAGGTCTGTATGG) and Hs_pex3-EagI (CGCGGCCGTTTCTCCAGTTGC). PCR product was cut with BglII and EagI and ligated into Myc in pUAST cut with the same enzymes.

#### pJM630 (dPex3-His_6_ in pET28a(+))

A region of dPex3 lacking the transmembrane domain was PCR amplified from a cDNA clone (DGRC #LD41491) using the oligos NdeI-PEX3 (GACATATGCGGCGATTCGTGG) and Dm_Pex3-NotI (CGGCGGCCGCAGCGGAGCTAAAGCTTTCG). PCR product was cut with NdeI and NotI and ligated into pET28a(+) cut with the same enzymes.

### Drosophila Stock Construction

pJM573 and pJM623 were injected into w^−^ embryos by Genetivision (Houston, TX). Injected flies were backcrossed twice to *w^−^* flies and multiple insertions were mapped to the chromosome. pJM875 and pJM877 were injected into VK37 and VK31 embryos by Genetivision (Houston, TX). Injected flies were backcrossed twice to w^−^ flies and stocks carrying insertions on chromosome two (VK37) and three (VK31) were established.

Independent P-element excision events using the P{Epgy2}pex3^EY19206^ line were screened for gene deletion events by PCR [67], [68]. One imprecise excision allele, *pex3^2^*, carries a 789 bp deletion (3L: 15, 137, 379.15, 138, 166) covering the first non-coding exon, intron one, and part of exon two including the translational start site and 285 bp of the coding sequence.

A P[acman] BAC CH322-17C13 which contained the entire pex3 locus was selected and obtained from P[acman] resources [69], [70]. Transgenic flies were then generated using PhiC31 integrase-mediated by injection into the y[1]w[1118]; PBAC{y[+]-attP}VK00037 strain which facilitated introduction of the genomic fragment onto the 2^nd^ chromosome [69]. This transgenic genomic fragment rescued the larval lethality in the *pex3^2^* homozygotes.

### Antibody Production and Immunoblots

pJM630 was expressed in *E. coli* BL21(DE3). Soluble Pex3-His_6_ was purified by nickel affinity chromatography and used as antigen to produce rabbit polyclonal antibodies (Cocalico). Anti-Pex3 serum was immunodepleted against total protein extract from *pex3^2^* mutant larvae immobilized on nitrocellulose and affinity purified using recombinant Pex3-His_6_ immobilized on nitrocellulose.

Total protein was extracted from ∼3 wandering 3^rd^ instar larvae that were collected fresh or frozen in liquid nitrogen and stored at −80°C. Larvae were homogenized in lysis buffer (25 mM HEPES pH 7.4, 100 mM KCl, 2 mM 2-mercaptoethanol, 1% triton X-100, 2 mM EDTA pH 8.0, Roche complete protease inhibitor). Total protein was quantified by Bradford assay and normalized prior to loading on homemade 4/12% polyacrylamide gels. Gels were transferred to nitrocellulose and probed with anti-Pex3 serum (1∶1000 dilution). Goat anti-Rabbit HRP- conjugated secondary antibodies (Rockland) were used at a 1∶10,000 dilution. Secondary antibodies were visualized by chemiluminescence using LumiGLO (Cell Signaling). Membranes were imaged on a LAS-4000 imager (FujiFilm) and band intensities were quantified in ImageJ.

### Imaging

Wandering 3^rd^ instar larvae were dissected in PBS, fixed in 4% formaldehyde, washed in PBS, and mounted in vectashield mounting media with DAPI (Vector Labs). Images were collected on a Zeiss LSM 510 confocal microscope. GFP was excited with a 488 nm argon laser and a HFT 488 primary dichroic. GFP emission was filtered with NFT 490 and BP 500–550 IR filters before collection. DAPI was excited with a chameleon two photon laser (Coherent) at 720 nm and a HFT KP 650 primary dichroic. DAPI emission was filtered with a 480–520 IR filter before collection.

Embryos were collected on grape juice plates, dechorionated, fixed, and mounted in vectashield with DAPI. Images were collected on a Zeiss Axioplan2 epifluorescent microscope. Images were deconvoluted with MetaMorph (Molecular Devices).

Wings were removed from adult flies in ethanol, mounted in euparal, and incubated overnight at 37°C. Wings, larvae, and pupal cases were imaged on a Leica MSV269 stereoscope.

### Lipidomics

*pex3^2^* and *pex3^rev^* flies were reared on cornmeal/molasses media and wandering 3^rd^ instar larvae were collected for analysis. Glycerophospholipids from homogenized *Drosophila* larvae of different genotypes were extracted using a modified Bligh and Dyer procedure [71]. Briefly, each sample was homogenized in 800 µL of ice-cold 0.1 N HCl∶CH_3_OH(1∶1) by vortexing for one minute at 4°C. Suspension was then vortexed with 400 µL of cold CHCl_3_ for one minute at 4°C and the extraction proceeded with centrifugation (5 min, 4°C, 18,000×g) to separate the two phases. Lower organic layer was collected and solvent evaporated. The resulting lipid film was dissolved in 100 µL of isopropanol∶hexane∶100 mM NH_4_COOH(aq) 58∶40∶2 (mobile phase A). Quantification of glycerophospholipids was achieved by the use of an LC-MS technique employing synthetic odd-carbon diacyl and lysophospholipid standards. Typically, 200 ng of each odd-carbon standard was added per sample. Glycerophospholipids were analyzed on an Applied Biosystems/MDS SCIEX 4000 Q TRAP hybrid triple quadrupole/linear ion trap mass spectrometer (Applied Biosystems, Foster City, CA, USA) and a Shimadzu high pressure liquid chromatography system with a Phenomenex Luna Silica column (2×250 mm, 5-µm particle size) using a gradient elution as previously described (Ivanova et al., 2007; Myers et al., 2011). The identification of the individual species, achieved by LC-MS/MS, was based on their chromatographic and mass spectral characteristics. This analysis allows identification of the two FA moieties but does not determine their position on the glycerol backbone (*sn-1* versus *sn-2*). Neutral lipids (DAG and TAG) were extracted by homogenizing weighed larvae samples in the presence of internal standards (300 ng 24∶0 DAG and 600 ng 42∶0 TAG) in 2 mL 1X PBS and extracting with 2 mL ethyl acetate∶trimethylpentane (25∶75). After drying the extracts, the lipid film was dissolved in 1 mL hexane∶isopropanol (4∶1) and passed through a bed of Silica gel 60 Å to remove remaining polar phospholipids. Solvent from the collected fractions was evaporated and lipid film was redissolved in 90 µL 9∶1 CH_3_OH∶CHCl_3_, containing 10 µL of 100 mM CH_3_COONa for MS analysis essentially as described (Lord et al., 2012). Samples were analyzed in triplicates and lipids are presented as pmol/mg weight for neutral lipids and pmol/mg protein for glycerophospholipids.

### Larval Starvation

Approximately 50 mating pairs were placed on 6 cm grape juice (20% grape juice, 2.4% agar, 2% ethanol, 1% acetic acid) plates at 25°C in the dark. Embryos were collected for 2 hours and incubated at 25°C. 66±1 hr old larvae were placed in 6 cm petri dishes without vents (Nunc, #150326) on a 3 cm square piece of Whatman blotting paper (GB004) soaked with PBS. Plates were wrapped with parafilm and incubated at 25°C in a humid chamber. Surviving larvae were counted every 24 hours.

### Climb Test

Adult flies were placed in 13×100 mm test tubes and gently vortexed. Video was captured with a Canon SD750 digital camera until the flies reached the top of the vial. The time required to crawl 5 cm was obtained from each video using VirtualDub (http://www.virtualdub.org).

## Supporting Information

Figure S1 ***pex3*** **knockdown causes aberrant lipid metabolism.** Total lipid extracts from wandering 3^rd^ instar larvae were analyzed by mass spectrometry at the Kansas lipidomics research center. (A) MS analysis of larval lipids shows that the levels of polar lipids are unchanged in larvae with reduced Pex3 (*Da^G32^>pex3.IR,dcr*) compared to controls (*Da^G32^>lacZ*). Diacylglycerol (DAG) levels are reduced and triacylglycerol (TAG) levels are elevated in larvae with reduced Pex3 compared to controls. (B) Larvae with reduced Pex3 also have elevated longer acyl chain length and reduced shorter acyl chain length TAG species.
(TIF)

Figure S2 **Neuronal and muscle morphology are unaltered in** ***pex3*** **mutants.** Larvae were dissected, fixed, and stained with Alexa 594-conjugated anti-HRP antibodies. Boutons at muscles 6 and 7 were visualized in *pex3^rev^* control (A) and *pex3^2^* mutant larvae (B) by fluorescence confocal microscopy. Larvae were also dissected, fixed, and stained with Alex 647-conjugated Phalloidin. One hemisegment was visualized in *pex3^rev^* control (C) and *pex3^2^* mutant larvae (C) by fluorescence confocal microscopy. In C and D, anterior is to the left and the ventral midline is to the bottom. The scale bar for A and B is 10 µm and the scale bar for C and D is 50 µm.
(TIF)

Figure S3 **Excitatory junction potentials (EJPs) at the neuromuscular junction are unaltered in** ***pex3*** **mutants.** Larvae were dissected in HL3.1 [72] with 0.8 mM CaCl_2_. Peripheral nerves were cut and stimulated with a suction electrode. Muscle recordings were taken from muscle 6 in abdominal segments A3, A4, or A5. (A) Representative traces for *pex3^rev^* control and *pex3^2^* mutant larvae show very similar EJP amplitudes. (B) Average EJP amplitudes ± standard error of the mean of *pex3^rev^* control (39.8±4.3 mV) and *pex3^2^* mutant larvae (35.4±1.9 mV) were not significantly different (*P* = 0.38, Student’s t-Test).
(TIF)

Video S1 **Peroxisome loss in muscles impairs locomotion.** Adult flies with reduced pex3 in muscles (*Mef2>pex3.IR, dcr*, right vial) climb slower and spend more time idle than control flies (*Mef2>dcr*, left vial).
(MOV)
