# Supplementary figures and images for: Peroxisomes Are Required for Lipid Metabolism and Muscle Function in Drosophila melanogaster
Source: PLoS One. 2014 Jun 19;9(6):e100213. doi: 10.1371/journal.pone.0100213 (PMC4063865; doi:10.1371/journal.pone.0100213)

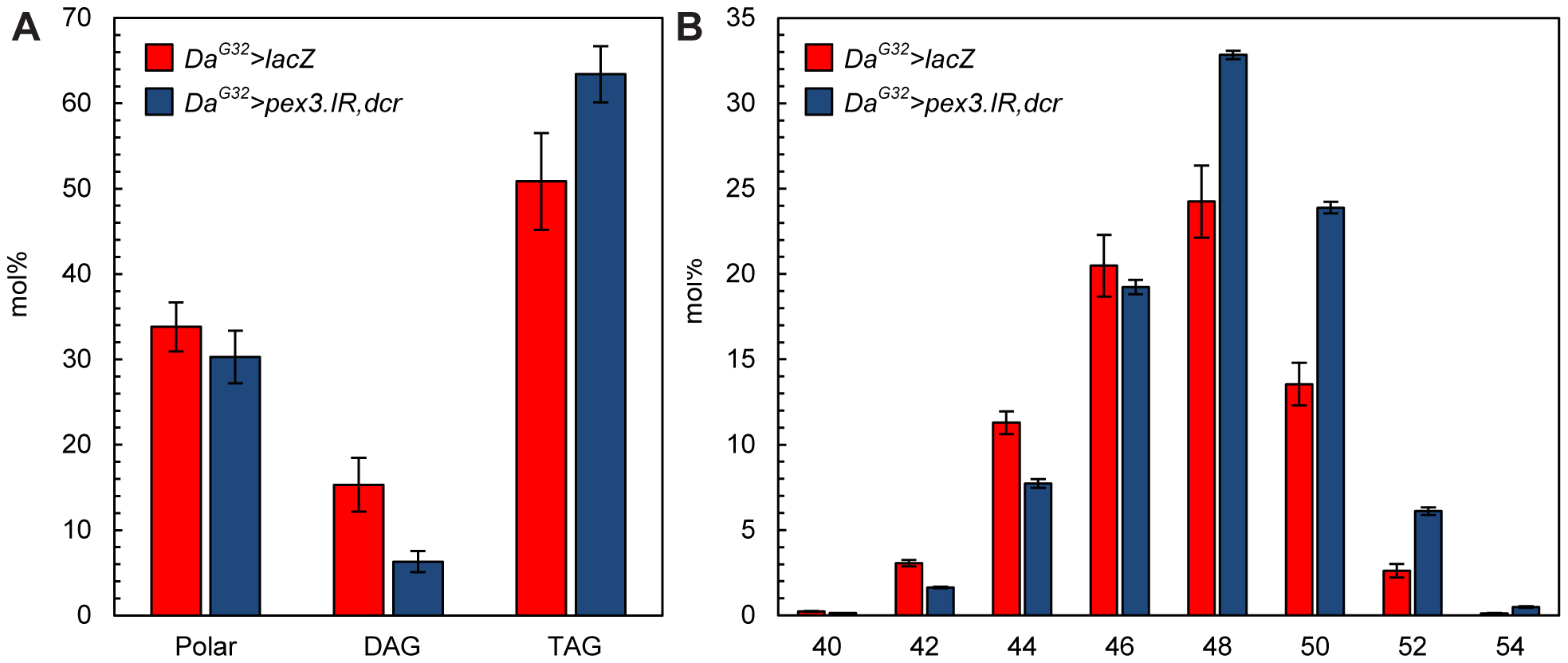

Supplement: Figure S1 — pex3 knockdown causes aberrant lipid metabolism. Total lipid extracts from wandering 3rd instar larvae were analyzed by mass spectrometry at the Kansas lipidomics research center. (A) MS analysis of larval lipids shows that the levels of polar lipids are unchanged in larvae with reduced Pex3 (DaG32>pex3.IR,dcr) compared to controls (DaG32>lacZ). Diacylglycerol (DAG) levels are reduced and triacylglycerol (TAG) levels are elevated in larvae with reduced Pex3 compared to controls. (B) Larvae with reduced Pex3 also have elevated longer acyl chain length and reduced shorter acyl chain length TAG species. (TIF) [file pone.0100213.s001.tif]

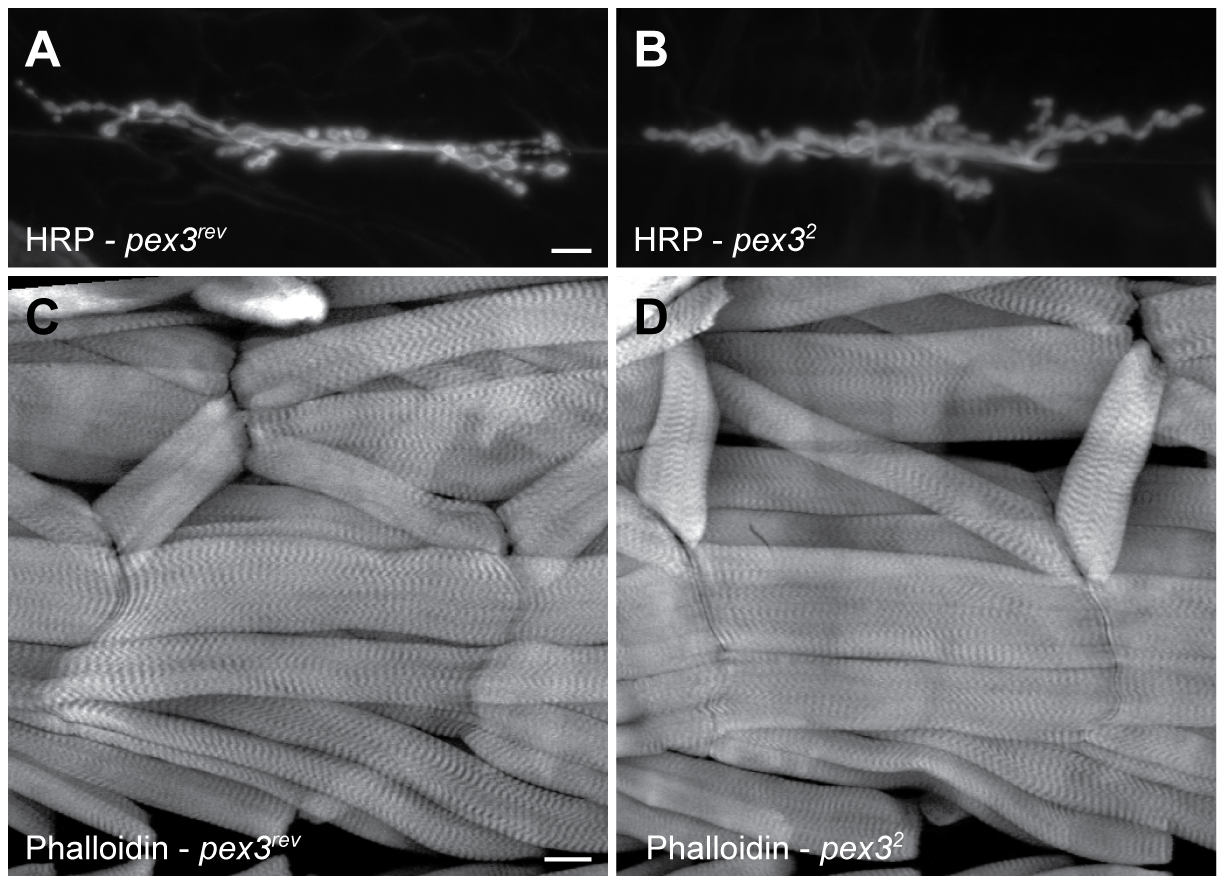

Supplement: Figure S2 — Neuronal and muscle morphology are unaltered in pex3 mutants. Larvae were dissected, fixed, and stained with Alexa 594-conjugated anti-HRP antibodies. Boutons at muscles 6 and 7 were visualized in pex3rev control (A) and pex32 mutant larvae (B) by fluorescence confocal microscopy. Larvae were also dissected, fixed, and stained with Alex 647-conjugated Phalloidin. One hemisegment was visualized in pex3rev control (C) and pex32 mutant larvae (C) by fluorescence confocal microscopy. In C and D, anterior is to the left and the ventral midline is to the bottom. The scale bar for A and B is 10 µm and the scale bar for C and D is 50 µm. (TIF) [file pone.0100213.s002.tif]

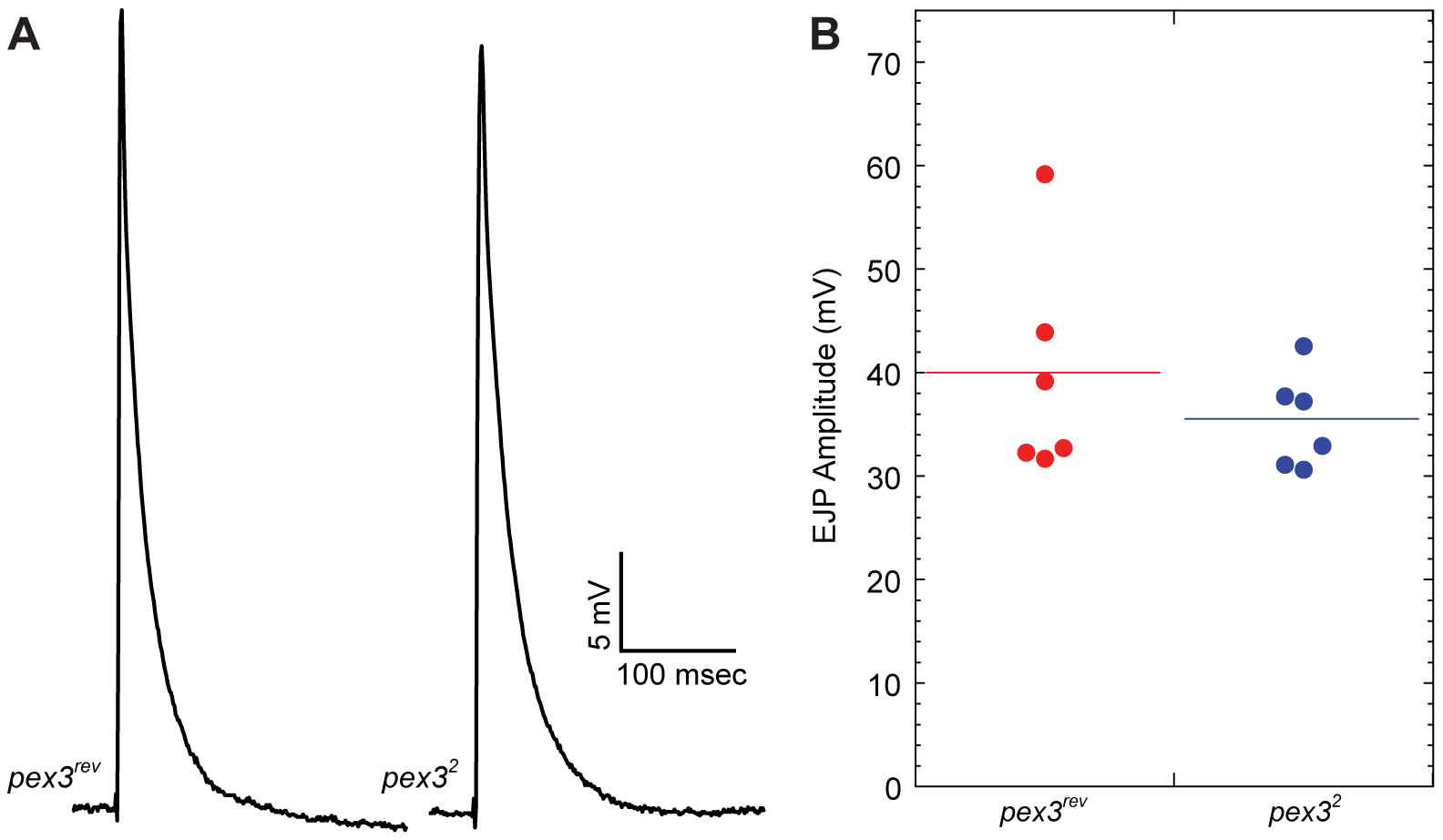

Supplement: Figure S3 — Excitatory junction potentials (EJPs) at the neuromuscular junction are unaltered in pex3 mutants. Larvae were dissected in HL3.1 [72] with 0.8 mM CaCl2. Peripheral nerves were cut and stimulated with a suction electrode. Muscle recordings were taken from muscle 6 in abdominal segments A3, A4, or A5. (A) Representative traces for pex3rev control and pex32 mutant larvae show very similar EJP amplitudes. (B) Average EJP amplitudes ± standard error of the mean of pex3rev control (39.8±4.3 mV) and pex32 mutant larvae (35.4±1.9 mV) were not significantly different (P = 0.38, Student’s t-Test). (TIF) [file pone.0100213.s003.tif]
